# The complete chloroplast genome sequence of *Populus davidiana* Dode

**DOI:** 10.1080/23802359.2016.1219634

**Published:** 2016-09-07

**Authors:** Mi Na Choi, Muho Han, Hong-Seog Park, Min-Young Kim, Ji-Seon Kim, Yoon-Jeong Na, Seung-Woo Sim, Eung-Jun Park

**Affiliations:** a Division of Forest Biotechnology, National Institute of Forest Science, Suwon, Korea;; b GnC Bio Co., Daejeon, Korea

**Keywords:** Chloroplast genome, *Populus davidiana* Dode, phylogenetic analysis

## Abstract

The complete chloroplast genome sequence of *Populus davidiana* Dode was determined in this study. The cpDNA was 155,853 bp in length, containing a pair of inverted repeats (IRs) of 27,571 bp each separated by a large and small single copy (LSC and SSC) regions of 84,127 bp and 16,584 bp, respectively. The cpDNA contained 130 genes, including 85 protein-coding genes, 8 ribosomal RNA genes and 37 transfer RNA genes. Phylogenetic analysis indicated *P. davidiana* was mostly close to *Populus tremula*, widely distributed in Europe and *Populus tremula × alba*.

*Populus davidiana* Dode (Korean aspen) is mainly distributed in North-Eastern Asia including Korea, China, Mongolia, and Far East Russia. It is known that morphologically and genetically related to *Populus tremula* L. and *Populus tremuloides Michx*. (Noh et al. 1997; Koo et al. 2007; Lee et al. 2011). In this study, we characterized the complete chloroplast genome sequence of *P. davidiana* for species identification and phylogenetic analysis. The annotated genome sequence has been deposited into Genbank under the accession number KX306825.

A plant material of *P. davidiana* (Accession number, Odea 19) was collected in 2015 from Kyungbuk-Youngju clonal plantation (36°49′N 128°31′E), Republic of Korea. This clone was originally collected from Mt Odae (37°47′N 128°32′E) in Republic of Korea and planted in 1992 to test the growth performance. This clone was selected as a superior clone based on mean 12-year stem volume and growth stability analysis (Noh et al. 1997; Koo et al. 2007). Total genomic DNA was extracted from fresh leaves of a single individual using the DNeasy Plant Mini Kit (Qiagen, Valencia, CA) and used for the three independent libraries for DNA sequencing; shotgun library for 454 GS FLX and 3kb and size free mate-pair libraries for Illumina Hiseq 2500. The whole-genome sequencing was conducted by GnC Bio Co. (Daejeon, South Korea). A total of 1021 M raw reads were retrieved, and quality-trimmed with default parameters using SOAPec v2.03 (Beijing Genomics Institute), Cutadapt (TU Dortmund, Science for Life Laboratory), DeconSeq-standalone v0.4.3 and Sickle v1.33 (GitHub, Inc.). The resultant 935 M trimmed reads were then used for the chloroplast genome reconstruction using the PLATANUS assembler, with that of the congener *Populus trichocarpa* (Genbank: EF489041.1). A total of 44,361,740 individual chloroplast reads generated an average coverage of ∼32,770×. The annotation was performed in Blast and DOGMA by comparing with the chloroplast genome of *P. trichocarpa* (EF489041.1) and *P. tremula* (Genbank: KP861984.1).

The circular genome is 155,853 bp in size, and comprises a pair of inverted repeat (IR) regions of 27,571 bp each, a large single-copy (LSC) region of 84,127 bp, and a small single-copy (SSC) region of 16,584 bp. The chloroplast genome contains 130 genes, including 85 protein-coding genes (77 PCG species), 8 ribosomal RNA genes (4 rRNA species) and 37 transfer RNA genes (30 tRNA species).

Complete chloroplast genome sequence of *P. davidiana* was aligned together with complete chloroplast genome sequences of nine other *Populus* species and *Salix interior* as an outgroup. Based on this alignment, the evolutionary history was generated by UPGMA method of MEGA 7 using 1000 bootstrap replicates (Felsenstein 1985; Kumar et al. 2016). Branches corresponding to partitions reproduced in less than 50% bootstrap replicates are collapsed. The percentage of replicate trees in which the associated taxa clustered together in the bootstrap test (1000 replicates) is shown next to the branches (Felsenstein 1985). The evolutionary distances were computed by Maximum-Composite Likelihood (MCL) method and are in the units of the number of base substitutions per site (Tamura et al. 2004). All *Populus* chloroplast genome sequences were clustered together. Within *Populus* cluster, *P. davidiana* was closely related to *P. tremula* (European aspen) and *P. tremula × P. alba* (clone 717-1B4) (Figure 1).

**Figure 1.**
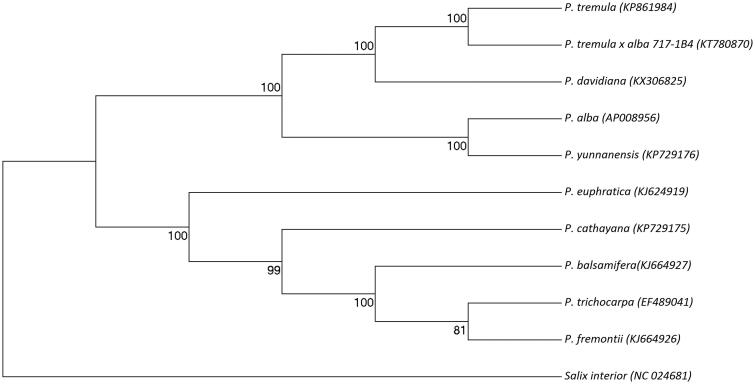
Phylogenetic tree of the complete chloroplast genome sequence of *Populus davidiana* with other 10 species. The tree was constructed using UPGMA method with MCL method and bootstrap support values (%) from 1000 replicates are shown above branches. Genbank accession numbers: *P. davidiana* (KX306825), *P. tremula* (KP861984), *P. tremula × P. alba* (KT780870), *P. alba* (AP008956), *P. yunnanensis* (KP729176), *P. euphratica* (KJ624919), *P. cathayana*(KP929175), *P. balsamifera* (KJ664927), *P. trichocarpa* (EF489041), *P. fremontii* (KJ664926), and *Salix interior* (NC024681).
